# Prognostic role of the systemic immune-inflammation index and pan-immune-inflammation value in acute methanol poisoning

**DOI:** 10.55730/1300-0144.6050

**Published:** 2025-07-26

**Authors:** Hatice ŞAHİN, Gülay ULUSAL OKYAY, Fatma AYERDEN EBİNÇ, Fevzi Coşkun SÖKMEN, Emre YAŞAR, Gülşen AKÇAY, Mehmet Deniz AYLI

**Affiliations:** 1 Division of Nephrology, Department of Internal Medicine, Etlik City Hospital, Ankara, Turkiye; 2 Department of Internal Medicine, University of Health Sciences, Gülhane Training and Research Hospital, Ankara, Turkiye; 3 Department of Emergency Medicine, Etlik City Hospital, Ankara, Turkiye

**Keywords:** Inflammation, methanol, poisoning, mortality

## Abstract

**Background/aim:**

Acute methanol poisoning (MP) poses a significant public health challenge, with inflammation increasingly recognized as a key factor in its pathophysiology. Identifying accessible and reliable prognostic biomarkers could help enhance clinical outcomes. This study aimed to assess the prognostic value of the systemic immune-inflammation index (SII) and the pan-immune-inflammation value (PIV), measured upon emergency department admission, in predicting in-hospital mortality in patients with acute MP.

**Material and methods:**

This retrospective study included patients diagnosed with acute MP at two tertiary care centers in Ankara, Türkiye: University of Health Sciences Dışkapı Yıldırım Beyazıt Education and Research Hospital (1 January 2015 to 1 October 2022) and Etlik City Hospital (1 October 2022 to 11 March 2025). Demographic, clinical, and laboratory data, along with treatment details and outcomes (discharge or inhospital death), were systematically recorded.

**Results:**

A total of 76 patients were included, of whom 92.1% were male, with a mean age of 49.0 ± 12.4 years. During hospitalization, 48.6% (n = 37) died. Both SII and PIV values at admission were significantly higher in nonsurvivors (p < 0.001 for SII; p = 0.031 for PIV). In multivariate Cox regression analysis, higher SII (HR: 2.44; 95% CI: 1.05–5.67; p = 0.034) and PIV (HR: 2.08; 95% CI: 1.05–4.13; p = 0.030) were independently associated with increased risk of mortality. Receiver operating characteristic (ROC) analysis showed an AUC of 0.750 (95% CI: 0.649–0.865) for SII, with an optimal cutoff of 665.6 (sensitivity: 50%; specificity: 46%), and an AUC of 0.640 (95% CI: 0.519–0.769) for PIV, with an optimal cutoff of 512.5 (sensitivity: 53%; specificity: 47%).

**Conclusion:**

SII and PIV measured at hospital admission may have potential prognostic value in predicting inhospital mortality in patients with acute MP.

## 1. Introduction

Methanol is a widely used, toxic, water-soluble solvent commonly found in industrial products. Significant oral ingestion, often from illicit or homemade alcohol, can lead to severe poisoning. After ingestion, it is metabolized first to formaldehyde and then to formic acid, the main toxic metabolite responsible for metabolic acidosis, cardiovascular instability, ocular toxicity, and neurological damage. The clinical presentation typically includes headache, gastrointestinal symptoms, visual disturbances, and altered mental status, with symptoms potentially appearing up to 72 h after exposure depending on the dose and route. Diagnosis usually depends on elevated serum methanol levels, presence of high anion gap metabolic acidosis, and evidence of end-organ damage [1,2]. However, early diagnosis is difficult due to incomplete histories, patients’ reluctance to disclose exposure because of legal concerns, and limited access to rapid methanol testing. Even when available, test results may take days, thereby delaying prompt treatment.

The incidence of acute methanol poisoning (MP) has risen during the COVID-19 pandemic, highlighting its growing impact on global health [3,4]. Due to the high morbidity and mortality associated with MP, research efforts have increasingly aimed to clarify its underlying mechanisms and identify prognostic markers to enable early diagnosis and improve clinical outcomes [1,2,3,5]. A significant factor contributing to methanol-induced toxicity is the interaction between oxidative stress and inflammation, which leads to tissue damage and worsens prognosis. Formic acid, a byproduct of methanol metabolism, inhibits mitochondrial cytochrome oxidase, resulting in cellular hypoxia and subsequent activation of oxidative and inflammatory pathways [6,7]. Experimental studies have shown that MP disrupts the hypothalamic-pituitary axis, increases oxidative stress, and alters immune responses, including changes in corticosterone levels. Additionally, the upregulation of markers such as 8-hydroxy-2′-deoxyguanosine, tumor necrosis factor-α, nuclear factor-kappa B, and interleukin-1β in optic nerve tissue, along with leukotriene-mediated neuroinflammation observed in brain injury models, highlights the role of inflammation in methanol-related cellular damage [6–9].

Although clinical data on this topic are also limited, a few studies have reported that certain inflammatory biomarkers such as red blood cell distribution width (RDW), neutrophil-to-lymphocyte ratio (NLR), and platelet-to-lymphocyte ratio (PLR) may be associated with poor clinical outcomes, including the need for mechanical ventilation, increased mortality, and vision loss [1,3]. However, these markers capture only partial aspects of the inflammatory response. The systemic immune-inflammation index (SII) and the pan-immune-inflammation value (PIV) are relatively novel composite indices that integrate multiple dimensions of immune and inflammatory activity. These indices have shown prognostic significance in a variety of acute and chronic conditions such as malignancies, aortic dissection, myocardial infarction, ankylosing spondylitis, and vasculitis [10–15]. Nevertheless, their clinical utility in acute MP has not been investigated yet.

This study aimed to assess the prognostic value of SII and PIV, measured upon admission to the emergency department, in predicting inhospital mortality among patients with acute MP.

## 2. Materials and methods

This retrospective observational study was approved by the Local Ethics Committee of Etlik City Hospital (Date: 12 March 2025; approval no: 2025–0276). The Institutional Review Board granted a full waiver of informed consent. All procedures were conducted in accordance with the Declaration of Helsinki.

**Figure 1:**
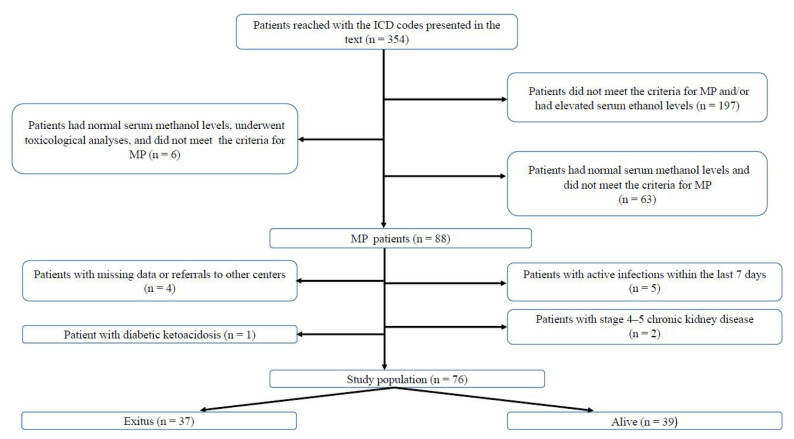
Flowchart of the study population. **Abbreviations:** ICD; international classification of diseases, MP; methanol poisoning.

Data were collected by reviewing records from Health Sciences University Dışkapı Yıldırım Beyazıt Education and Research Hospital (1 January 2015 to 1 October 2022) and Etlik City Hospital (1 October 2022 to 11 March 2025), both of which are part of the same healthcare network. The following International Classification of Diseases (ICD) codes were used to identify potential cases from the hospitals’ electronic record systems: T51 (toxic effects of alcohol), T51.0 (toxic effects of ethanol), T51.1 (toxic effects of methanol), T51.9 (toxic effects of alcohol, unspecified), Z72.1 (alcohol use), F10 (mental and behavioral disorders due to alcohol use), and Y91.9 (alcohol involvement). An initial cohort of 354 patients was identified. Eighty-eight patients were diagnosed with MP based on medical history, physical examination, and laboratory findings. Among these, patients with an active infection within the last 7 days (n = 5), missing data or referral to other centers (n = 4), stage 4–5 chronic kidney disease (n = 2), or diabetic ketoacidosis (n = 1) were excluded. None of the patients had a history of blood transfusion in the past month, malignancy, hematologic or rheumatologic disease, immunosuppressive therapy, or chronic liver disease. Consequently, a study cohort of 76 patients was formed (Figure 1).

### 2.1. Diagnosis of methanol poisoning

Serum methanol levels are not always measurable in our healthcare facilities, and when tested, results often take several days to become available. Therefore, at our center, MP diagnosis is based on a history or strong evidence of methanol ingestion; clinical symptoms such as altered consciousness, impaired coordination, vomiting, abdominal pain, and blurred or decreased vision; along with the presence of at least two of the following laboratory abnormalities: pH < 7.3, bicarbonate (HCO_3_^−^) < 20 mmol/L, and elevated anion gap on blood gas analysis. In cases where serum methanol concentrations are measured, diagnosis is confirmed by elevated methanol levels.

### 2.2. Patient data

Patient data—including age, sex, history of illicit alcohol use, type of intoxication, comorbidities, and vital signs at initial presentation to the emergency department—were systematically recorded. Initial laboratory tests performed upon admission—complete blood count, biochemical analyses, blood gas analysis, and serum methanol levels (if available)—were documented. Additional data collected included details of hospitalization (emergency department, inpatient wards, intensive care units [ICU]), treatments administered, length of hospital stay, need for mechanical ventilation (MV), and clinical outcomes (discharge or death). For patients undergoing hemodialysis (HD), the number and duration of sessions were recorded. Ophthalmological evaluations for visual disturbances and steroid treatment recommendations were also noted. Patients were followed from emergency department presentation until discharge or death.

### 2.3. Laboratory assays

**Figure 2:**
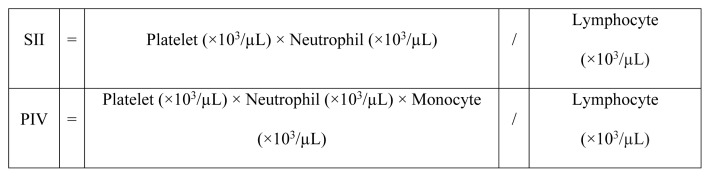
SII and PIV score calculation formulas. **Abbreviations:** SII; systemic immune-inflammation index, PIV; pan-immune-inflammation value.

Complete blood counts were analyzed using the Sysmex XN-1000 Hematology Analyzer (Sysmex Corp., Kobe, Japan). Serum creatinine levels were measured with the Roche Cobas c702 device (Roche Diagnostics GmbH, Mannheim, Germany), utilizing the Jaffé method. The estimated glomerular filtration rate (eGFR) was calculated using the Chronic Kidney Disease Epidemiology Collaboration (CKD-EPI) creatinine equation [16]. Serum methanol levels were determined by gas chromatography-mass spectrometry (GC-MS) using Agilent GC-FID and MS5977E instruments (Agilent Technologies Inc., Santa Clara, CA, USA). Blood gas analysis was performed using Siemens Rp500 and RL1265 devices (Siemens Healthineers, Erlangen, Germany). The anion gap was determined by the difference between serum sodium concentration (Na^+^) and the sum of serum chloride (Cl^−^) and HCO_3_^−^ using the formula [Na^+^ − (Cl^−^ + HCO_3_^−^)]. The SII and PIV were calculated according to the formulas presented in Figure 2 [17,18].

### 2.4. Treatment protocols

Management of acute MP at our centers usually follows the American Academy of Clinical Toxicology guidelines [19]. Treatment is tailored according to symptom severity and may include ethanol, fomepizole, sodium bicarbonate, folate or leucovorin, corticosteroids, HD, and continuous venovenous hemodiafiltration (CVVHD). HD is initiated in patients presenting with high anion gap metabolic acidosis (pH ≤ 7.30), evidence of end-organ damage such as visual disturbances or neurological impairment, or hemodynamic instability refractory to conservative management. CVVHD is preferred for hemodynamically unstable patients. Patients with pH < 7.3 receive an initial intravenous bolus of sodium bicarbonate (1–2 mEq/kg), followed by continuous infusion to maintain pH > 7.3. Prednisolone is administered to those with visual impairment or retinal involvement.

### 2.5. Statistical analyses

Statistical analyses were performed using SPSS version 24.0 (IBM Corp., Armonk, NY, USA). The distribution of continuous variables was evaluated with the Kolmogorov–Smirnov test. Normally distributed variables were compared using one-way analysis of variance (ANOVA) and presented as mean ± standard deviation (SD). Nonnormally distributed data were analyzed with the Mann–Whitney U test and expressed as median with interquartile range (IQR). Categorical variables were compared using the chi-square test or Fisher’s exact test, and results are shown as counts and percentages. Univariate and multivariate Cox proportional hazards regression analyses were conducted to identify factors associated with mortality. Variables with p < 0.25 in univariate analysis were entered into the multivariate model. Due to the limited sample size and event numbers, multivariate analyses were adjusted for age and sex. Log-transformed values were used for nonnormally distributed variables when appropriate. Hazard ratios (HR) with 95% confidence intervals (CI) are reported. The predictive performance of SII and PIV for mortality was assessed using receiver operating characteristic (ROC) curve analysis. Statistical significance was defined as p < 0.05.

## 3. Results

The baseline characteristics of all patients diagnosed with MP, as well as comparisons between survivors and nonsurvivors, are summarized in Table 1.

### 3.1. General characteristics of the study population

The study included 76 patients with a mean age of 49.03 ± 12.40 years; 92.1% were male. The mean arterial pressure (MAP) at emergency department admission was 94.41 ± 27.68 mmHg. Serum methanol levels were available for 53 patients (69.7% of the cohort), while ethanol levels were within the normal range in all patients. The median hospital stay was 8 days (IQR: 5.25–14). During follow-up, 63.2% required MV, and 48.6% (n = 37) died. Detailed findings are summarized in Table 1.

### 3.2. Comparison between survivors and nonsurvivors

Visual impairment was reported more frequently among survivors (p = 0.02). Nonsurvivors presented with significantly lower MAP at admission (p < 0.001) and required MV more often (p < 0.001). Compared to survivors, nonsurvivors had lower eGFR (p = 0.009), blood pH, and bicarbonate levels (both p < 0.001), alongside higher serum glucose, sodium, and potassium concentrations (p = 0.001, p = 0.001, and p = 0.004, respectively). They also showed elevated anion gap (p = 0.02), lactate (p < 0.001), and base deficit (p < 0.001). Both the SII and PIV were significantly higher in nonsurvivors (p < 0.001 and p = 0.031, respectively) (Table 1). Among the 53 patients with measured serum methanol levels, the median concentrations were 55.63 mg/dL (IQR: 29.43–137) in survivors and 103 mg/dL (IQR: 81.8–203) in nonsurvivors (p = 0.02).

### 3.3. Medical care of patients at hospital admission and during hospitalization

Among the patients, 92.1% (n = 70) received sodium bicarbonate, 76.3% (n = 58) were treated with ethyl alcohol, 35.5% (n = 27) received corticosteroids, and 5.3% (n = 4) were administered fomepizole. Conventional HD was performed in 96.1% (n = 73) of patients at admission and during follow-up, while 15.7% (n = 12) underwent both conventional HD and CVVHD. Intravenous calcium folinate was administered in only one case.

### 3.4. Cox regression analysis

**Figure 3:**
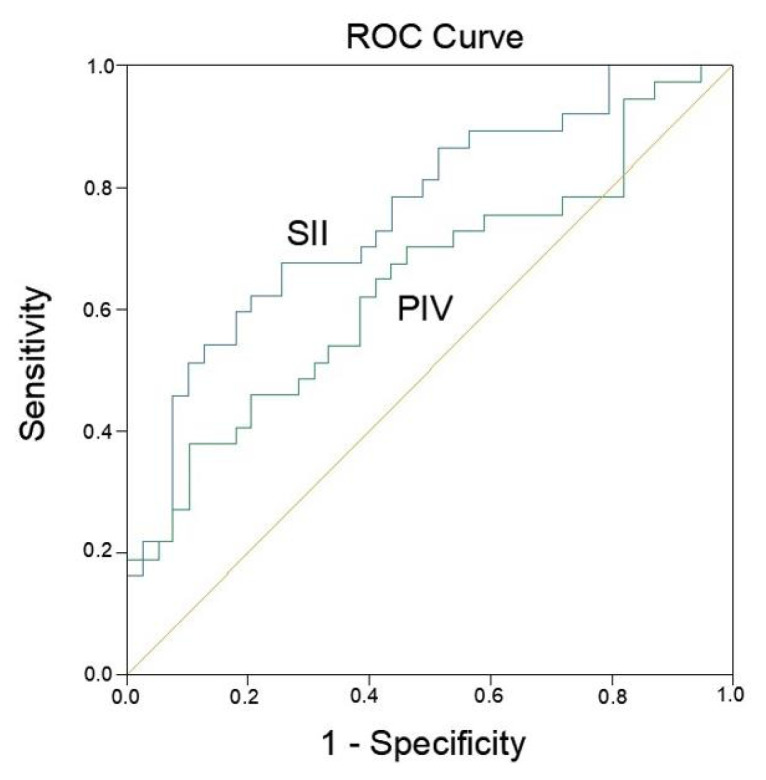
Receiver operating characteristic (ROC) curve of SII and PIV for predicting mortality in patients with methanol poisoning. SII: systemic immune-inflammation index [AUC: 0.75, p < 0.001, 95% CI (0.649–0.865)]; PIV: pan-immune-inflammation value [AUC: 0.644, p = 0.03, 95% CI (0.519–0.769)].

In univariate Cox regression analysis, variables significantly associated with mortality included sex, MAP, MV requirement, lactate, base deficit, pH, bicarbonate (HCO_3_^−^), SII, and PIV. In the multivariate model, independent predictors of inhospital mortality were identified as follows: lower MAP [HR: 0.98; 95% CI: 0.962–0.993; p = 0.006], MV requirement [HR: 13.15; 95% CI: 1.76–98.5; p = 0.01], higher serum glucose [HR: 1.003; 95% CI: 1.000–1.005; p = 0.007], elevated lactate [HR: 4.14; 95% CI: 1.16–14.90; p = 0.03], increased base deficit [HR: 1.07; 95% CI: 1.02–1.13; p = 0.008], decreased pH [HR: 0.20; 95% CI: 0.05–0.88; p = 0.02], lower HCO_3_^−^ [HR: 0.804; 95% CI: 0.674–0.958; p = 0.015], elevated SII [HR: 2.44; 95% CI: 1.05–5.67; p = 0.034], and elevated PIV [HR: 2.08; 95% CI: 1.05–4.13; p = 0.03] (Table 2). ROC analysis showed an area under the curve (AUC) of 0.750 (95% CI: 0.649–0.865) for SII, with an optimal cutoff of 665.56 (sensitivity 50%; specificity 46%), and an AUC of 0.640 (95% CI: 0.519–0.769) for PIV, with an optimal cutoff of 512.52 (sensitivity 53%; specificity 47%) (Figure 3).

## 4. Discussion

This study evaluated the clinical characteristics of patients with MP and explored the prognostic value of systemic inflammatory biomarkers in predicting inhospital mortality. Our findings indicated that the study population was predominantly composed of relatively young men with few comorbidities. Despite this, a substantial proportion required intensive interventions, including HD and MV, and the inhospital mortality rate approached 50%. Importantly, elevated admission levels of both SII and PIV were independently associated with increased mortality risk. Additional mortality predictors included markers of hemodynamic instability, hyperglycemia, and severe metabolic acidosis.

Previous experimental and clinical studies have demonstrated a clear association between MP and both oxidative stress and systemic inflammation, as evidenced at the tissue level and in peripheral blood markers [6–9]. Traditional inflammatory indicators such as RDW, NLR, and PLR have been linked to adverse outcomes in MP. For instance, elevated RDW has been associated with increased mortality and a greater need for MV, while high NLR and PLR values at admission correlate with worse inhospital outcomes [1,3]. However, evidence regarding more comprehensive inflammatory indices in MP remains limited. Composite markers that integrate multiple immune and inflammatory components may provide a broader and more accurate assessment of systemic inflammation. In this context, SII and PIV, which combine neutrophil, platelet, lymphocyte, and/or monocyte counts, offer a more holistic perspective compared to the aforementioned indices. According to the literature, SII was originally introduced to evaluate immune-inflammatory status and predict morbidity and mortality in cancer patients [20,21]. It has also been shown to reflect disease activity and predict poor outcomes in vasculitis [15]. Similarly, PIV, originally proposed by Fucà et al. as a prognostic tool in metastatic colorectal cancer, has been associated with increased mortality in various clinical settings, including vasculitis, myocardial infarction, and hypertension, and has demonstrated a nonlinear relationship with 28-day and 90-day mortality in sepsis [10,11,13,22,23]. The prognostic relevance of SII and PIV has also been explored in chronic inflammatory and autoimmune diseases [24,25]. These indices have shown promise in distinguishing disease activity and risk stratification in conditions such as sarcoidosis and idiopathic membranous nephropathy [18,26].

To the best of our knowledge, this is the first study to evaluate the prognostic significance of the SII and PIV in predicting inhospital mortality among patients with MP. Our findings demonstrate that elevated baseline levels of both SII and PIV are independently associated with increased mortality, underscoring their potential utility as accessible and cost-effective biomarkers for early risk stratification in acute MP. These results align with prior evidence linking systemic inflammation to adverse clinical outcomes in this population. Notably, ROC curve analysis revealed that SII may exhibit superior predictive performance compared to PIV. However, given the comparable values observed for both indices in our cohort and the lack of previous studies directly comparing them in the context of MP, further large-scale prospective research is warranted to validate the prognostic advantage of SII.

Accumulating evidence in the literature has identified several prognostic factors associated with poor outcomes in acute MP. These include hyperglycemia at admission [5,27], severe metabolic acidosis [19,28–33], elevated serum methanol concentrations [34–36], delayed presentation to the hospital [29,37], neurological complications such as coma or seizures [27–29,31,32,34,35], the need for MV [27,31,32,38], and low MAP [38,39]. Consistent with these reports, our study also identified low MAP, hyperglycemia, metabolic acidosis at admission, and the need for MV as significant independent predictors of inhospital mortality.

High anion gap metabolic acidosis is a hallmark of MP, primarily driven by the accumulation of formic and lactic acids [2,32,40]. Formic acid not only contributes to the acid load but also inhibits mitochondrial oxidative phosphorylation, thereby promoting anaerobic metabolism and elevated lactate production. Additionally, hemodynamic instability and impaired tissue perfusion may further augment lactate levels [2]. In our study, blood gas parameters—including pH, bicarbonate, base deficit, and lactate—were significantly associated with inhospital mortality and may serve as valuable markers for early identification of high-risk patients. However, the anion gap—despite being a commonly used parameter—was not significantly associated with mortality. This observation aligns with findings by Cömertpay et al. [41]. Several explanations are plausible, including the possibility of a normal anion gap in the early stages of methanol intoxication [2,42,43], heterogeneity in methanol exposure doses, and individual variability in hemodynamic response. These aspects warrant further exploration in future studies with more precise data on the timing and quantity of methanol ingestion.

Measurement of serum methanol levels provides critical prognostic information by directly reflecting the toxic burden in MP. However, limited availability of methanol assays in many healthcare centers restricts their routine clinical application. This situation contributed to the incomplete data in our cohort. Specifically, serum methanol concentrations were measured in 53 patients. In line with previous studies [34–36], we found that mortality was higher among those with elevated methanol levels. To our knowledge, this is the first study to report such findings from our country. Given its prognostic value, we recommend routine measurement of serum methanol levels in all suspected MP cases and emphasize the need for more rapid testing availability to support timely risk stratification and clinical decision-making.

Hyperglycemia may develop in patients with MP even in the absence of preexisting diabetes. In our cohort, median blood glucose levels were significantly higher in nonsurvivors compared to survivors, and hyperglycemia was identified as an independent predictor of inhospital mortality in multivariate analysis. This finding is consistent with a limited number of previous studies [5,27]. The underlying mechanisms of hyperglycemia in acute MP remain unclear, though potential contributors include the acute stress response and methanol-induced pancreatitis [5]. Moreover, the coexistence of hyperglycemia and metabolic acidosis at admission may mimic diabetic ketoacidosis, thereby complicating the diagnostic process, particularly in patients who are unable to provide a reliable medical history due to their clinical condition. Accurate differential diagnosis is essential and can be achieved through the absence of urinary ketones and the presence of elevated serum methanol levels. Recognizing this diagnostic challenge is critical for increasing clinical awareness and facilitating timely and appropriate treatment decisions.

Visual disturbances are a hallmark of MP and may occur either in isolation or alongside other systemic symptoms. These manifestations primarily result from the toxic effects of formate on the optic nerve and retinal epithelial cells [2,19,44,45]. In our study, 55.3% of patients reported visual disturbances, with a significantly higher prevalence among survivors than nonsurvivors. This difference likely reflects the greater capacity of survivors to communicate symptoms and undergo ophthalmologic assessment, whereas critically ill or intubated patients may have been underevaluated. These findings underscore the diagnostic significance of visual symptoms in MP and highlight the importance of prompt and thorough ophthalmologic evaluation, particularly in patients presenting with severe poisoning.

Intermittent HD is the preferred extracorporeal treatment modality for MP, as it efficiently removes methanol and its toxic metabolites while correcting acid-base disturbances [2,19,46]. Kute et al. demonstrated that early initiation of HD, in conjunction with adjunctive treatments such as bicarbonate and ethanol, significantly reduces mortality [32]. Peces et al. emphasized the importance of maintaining dialysate flow rates above 250 mL/min, using bicarbonate-enriched solutions supplemented with potassium and phosphorus, and prolonging dialysis duration to optimize clearance [46]. In settings where methanol levels cannot be measured promptly, an 8 h HD session is generally recommended [47]. Nevertheless, variations in dialysis practices and patient characteristics may contribute to different outcomes reported in the literature. For instance, one study reported that only 21.7% of patients received HD, with an average session duration of less than 3 h, yet a relatively low mortality rate of 11% was observed [36]. This finding, although seemingly at odds with the established benefits of HD, may reflect differences in case severity, early diagnosis, and local treatment protocols. In contrast, our cohort exhibited a markedly higher HD utilization rate. This may be explained by delayed availability of methanol test results, the presence of severe metabolic acidosis or organ dysfunction at presentation, or clinical deterioration despite initial conservative measures. Additionally, as a tertiary referral center, our institution likely admitted a greater proportion of critically ill patients requiring more aggressive intervention. In our study, the mean duration of HD sessions was approximately 4 h, which may reflect technical or institutional constraints. No significant differences in HD requirement or treatment duration were observed between survivors and nonsurvivors, consistent with the findings of Tümer et al. [39]. Although CVVHD was used for hemodynamically unstable patients, it is generally considered less effective than conventional intermittent HD. Taken together, these findings highlight the need for further research to define the optimal timing, duration, and modality of dialysis in the treatment of MP.

This study has several limitations. As a retrospective, cross-sectional analysis with a relatively small sample size, its ability to infer causal relationships is inherently limited. Serum methanol levels were not available for all patients in our cohort, and key markers such as the osmolar gap and C-reactive protein were largely missing. Furthermore, only baseline laboratory values at the time of emergency department admission were analyzed, without follow-up data to evaluate dynamic changes. The precise timing of methanol ingestion was also unknown, limiting the assessment of temporal relationships between exposure and clinical or laboratory findings. Despite these limitations, the study has several important strengths. To our knowledge, it is the first to demonstrate an independent association between SII, PIV, and inhospital mortality in MP. Additionally, it provides valuable insights into the inflammatory component of MP and represents one of the largest cohorts reported from our country, including the first dataset on measured serum methanol levels.

In conclusion, acute MP is a life-threatening toxicological emergency that predominantly affects relatively young individuals with few comorbidities, yet leads to considerable morbidity and mortality. Given that serum methanol levels, critical for definitive diagnosis and management, are often unavailable or delayed, there is a clear need for accessible prognostic markers. This study highlights the potential utility of the SII and PIV, which are inexpensive and easily derived from routine blood counts, for early risk stratification. Our findings underscore their prognostic value and further suggest a key role for acute inflammation in the pathophysiology of MP, laying the groundwork for future diagnostic and therapeutic strategies.

## Figures and Tables

**Table 1:** General characteristics of the study population and subgroups.

	Study population	Survivors	Nonsurvivors	p
	(n = 76)	(n = 39)	(n = 37)	
Age, years	49.0 ± 12.4	49.0 ± 12.1	49.0 ± 12.9	1.000
Sex, male (n, %)	70 (92.1)	39 (100)	31 (83.8)	**0.010**
Mean arterial blood pressure (mmHg)	94.4 ± 27.7	106.2 ± 22.5	81.9 ± 27.4	**<0.001**
Visual impairment (n, %)	42 (55.3)	27 (69.2)	15 (42.9)	**0.020**
Comorbidities (n, %)				
• Hypertension	15 (19.7)	8 (20.5)	7 (18.9)	0.540
• Diabetes mellitus	5 (6.6)	3 (7.7)	2 (5.4)	0.520
• Coronary artery disease	5 (6.6)	4 (10.3)	1 (2.8)	0.360
Mechanical ventilation (n, %)	48 (63.2)	12 (30.8)	36 (97.3)	**<0.001**
Length of stay, hospital (days)	8 (5.25–14)	9 (7–14)	7 (1.5–16.5)	0.100
Length of stay, ICU (days)	6 (3–10)	5 (3–8)	7 (1.5–16.5)	0.450
Conventional HD (n, %)	73 (96.1)	38 (97.4)	35 (94.6)	0.110
Conventional HD duration (hours)	4 (3–8)	4 (4–7)	8 (2–12)	0.190
**Laboratory findings**				
• Hemoglobin (g/dL)	15.8 ± 2.3	16.1 ± 2.2	15.4 ± 2.4	0.200
• White blood cell count (/μL) ×10^3^	11.69 (9.72–15.52)	11.58 (8.51–15.0)	11.83 (4.49–9.98)	0.290
• Platelet count (/μL) ×10^3^	250 (205–309)	83 (62–98)	229 (179–165)	0.360
• Glucose (mg/dL)	147 (108–224)	119 (95–160)	179 (132–268)	**0.001**
• Urea (mg/dL)	23 (19–36)	23 (18–40)	23 (20–32)	0.400
• Creatinine (mg/dL)	1.17 (0.97–1.44)	1.07 (0.95–1.42)	1.27 (1.07–1.50)	**0.040**
• eGFR (mL/min/1.73 m^2^)	72 (57–93)	83 (62–98)	64 (53–80)	**0.009**
• Sodium (mEq/L)	137.3 ± 3.3	136.1 ± 3.4	138.5 ± 2.7	**0.001**
• Potassium (mEq/L)	5.1 ± 1.1	4.7 ± 0.9	5.4 ± 1.2	**0.004**
• pH	6.93 ± 0.22	7.04 ± 0.17	6.82 ± 0.21	**< 0.001**
• Bicarbonate (mmol/L)	6.8 ± 2.4	7.7 ± 2.3	5.8 ± 2.1	**<0.001**
• Anion gap	25.0 ± 5.9	24.5 ± 5.9	27.6 ± 5.4	**0.020**
• Base deficit (mmol/L)	25.5 ± 6.5	23.0 ± 5.8	28.1 ± 6.2	**<0.001**
• Lactate (mmol/L)	6.5 (2.6–9.6)	3.0 (1.6–7.0)	8.3 (6.3–11.0)	**<0.001**
• SII (×10^3^)	920 (387–1578)	485 (269–1068)	1528 (605–2138)	**<0.001**
• PIV (×10^6^)	626 (324–1182)	478 (311–915)	757 (366–1443)	**0.031**

**Abbreviations:** CKD-EPI; Chronic Kidney Disease Epidemiology Collaboration formula, eGFR; estimated glomerular filtration rate, HD; hemodialysis, ICU; intensive care unit, SII; systemic immune-inflammation index, PIV; pan-immune-inflammation value. Significant values are shown in bold.

**Table 2:** Univariate and multivariate Cox regression analyses to determine factors affecting mortality.

	Univariate analysis		Multivariate analysis	
	HR (% 95 CI)	p	HR (% 95 CI)	p
Age	0.99 (0.93–1.02)	0.549		
Sex	3.1 (1.26–7.61)	**0.01**		
Mean arterial blood pressure (mmHg)	0.98 (0.96–0.99)	**0.002**	0.98 (0.962–0.993)	**0.006**
Hypertension	1.32 (0.32–5.56)	0.69		
Diabetes mellitus	1.32 (0.32–5.56)	0.69		
Coronary artery disease	2.97 (0.40–21.87)	0.283		
Visual impairment	1.84 (0.92–3.66)	0.08	1.9 (0.94–3.78)	0.070
Mechanical ventilation	14.59 (1.97–107.88)	**0.009**	13.15 (1.76–98.5)	**0.010**
Conventional HD	2.44 (0.575–10.365)	0.227	1.67 (0.371–7.51)	0.500
Conventional HD duration	1.20 (0.47–3.11)	0.70		
Hemoglobin (g/dL)	1.01 (0.89–1.144)	0.87		
White blood cell count (/μL)	1 (1–1)	0.88		
Glucose (mg/dL)	1.002 (1–1.005)	0.11	1.003 (1–1.005)	**0.007**
Urea (mg/dL)	0.98 (0.97–1.02)	0.14	0.983 (0.958–1.01)	0.220
Creatinine (mg/dL)	1.13 (0.55–2.32)	0.74		
eGFR (mL/min/1.73m^2^)	0.99 (0.98–1.06)	0.25	0.99 (0.97–1.01)	0.200
Sodium (mEq/L)	1.072 (0.975–1.179)	0.153	1.07 (0.972–1.185)	0.161
Potassium (mEq/L)	1.37 (0.99–1.88)	0.05	1.35 (0.978–1.860)	0.060
Bicarbonate (mmol/L)	0.783 (0.66–0.93)	**0.005**	0.804 (0.674–0.958)	**0.015**
Anion gap	1.08 (1–1.12)	0.05	1.04 (0.98–1.10)	0.150
Lactate (mmol/L)	4.86 (1.4–17.15)	**0.01**	4.14 (1.16–14.90)	**0.030**
Base deficit (mmol/L)	1.08 (1.03–1.132)	**0.003**	1.07 (1.02–1.126)	**0.008**
SII (×10^3^)	2.94 (1.214–7.134)	**0.017**	2.44 (1.05–5.67)	**0.034**
PIV (×10^6^)	2.415 (1.251–4.663)	**0.009**	2.08 (1.05–4.13)	**0.030**

**Abbreviations:** eGFR; estimated glomerular filtration rate, HD; hemodialysis, SII; systemic immune-inflammation index, PIV; pan-immune-inflammation value. The results are presented with hazard ratios (HRs) and 95% confidence intervals (CIs). Multivariate models were adjusted for age and sex. Significant values are shown in bold.
